# Beta-2 Glycoprotein I IgA Isotype: An Important Consideration in Secondary Hypertension

**DOI:** 10.7759/cureus.60560

**Published:** 2024-05-18

**Authors:** Thomas F Fusillo, Lydia Capicotto, Jaskirat Gill

**Affiliations:** 1 Internal Medicine, Icahn School of Medicine at Mount Sinai, New York, USA; 2 Medicine, Edward Via College of Osteopathic Medicine, Blacksburg, USA; 3 Critical Care, Icahn School of Medicine at Mount Sinai, New York, USA

**Keywords:** anti-cardiolipin antibody, autoimmune disorders, non-malignant hematology, antiphospholipid antibody, transient ischemic attacks, coagulation disorder, acute cva, cerebro-vascular accident (stroke), primary antiphospholipid antibody syndrome, secondary hypertension

## Abstract

Anti-beta-2 glycoprotein I antibodies are an important player in hypercoagulable states, including those that lead to antiphospholipid syndrome. Traditionally, assays have only detected IgG and IgM isotypes of this antibody. However, newer assays also detect the IgA isotype. The problem lies in the largely unknown significance of this IgA isotype. This paper describes a middle-aged male who presented with hypertensive emergency and was later found to have IgA anti-beta-2 glycoprotein I antibodies. He was treated with multiple anti-hypertensives, aspirin, and statin therapy. In addition to the case, we discuss the implications of this IgA isotype and how it may relate to antiphospholipid syndrome, despite not currently being included in the laboratory diagnostic criteria for the disease.

## Introduction

Beta-2 glycoprotein I (B2GI) is a soluble blood protein with multiple known roles, including hemostasis. It is also one of the main antigenic targets by autoantibodies in the condition antiphospholipid syndrome (APS). However, there is potential to uncover more of its physiology outside of APS [1]. Anti-beta-2 glycoprotein (anti-B2GI) is one of the three autoantibodies that are included in the diagnostic criteria for APS, along with antiphospholipid and anti-cardiolipin antibodies. However, the widely accepted diagnostic criteria for the condition, the Sapporo criteria, only recognizes the IgG and IgM isotypes of anti-B2GI [2]. Thus, the IgA isotype of anti-B2GI remains of little known clinical significance. This article discusses a case of hypertensive emergency that, upon further investigation, was positive for the IgA isotype of anti-B2GI and explains the relevant workup and current state of the literature on this topic.

## Case presentation

An African-American man in his mid-40s with hypertension presented to the emergency department of an urban tertiary care center with 5-6 months of worsening chest pain, headaches, and numbness in his feet. He had been living in the Caribbean islands for seven years and arrived back in the United States that day and came straight to the hospital. The chest pain, numbness, and headaches have all been intermittent. The chest pain is pressure-like and is concomitant with the headaches. The numbness is on the dorsal aspect of both feet, intermittent, and not always associated with his other symptoms. Additionally, he has been unable to get an erection nor wake up with an erection for the past two months.

While in the Caribbean, he was in and out of several clinics and hospitals and was found to be hypertensive each time and was intermittently taking a variety of anti-hypertensive medications including carvedilol, hydralazine, and labetalol. However, he stopped taking all his medications due to not seeing an improvement in symptoms. He received very limited workup while abroad due to a lack of healthcare resources.

On exam, the patient's blood pressure was 255/149 mmHg, with a heart rate of 85 beats per minute and an oxygen saturation of 99% on room air, and he was afebrile. His physical exam was grossly unremarkable with regular rate and rhythm without any murmurs, clear lung sounds, cranial nerves all intact, and strength full and symmetric, and he was alert and oriented to person, place, and time.

Investigations

**Figure 1:**
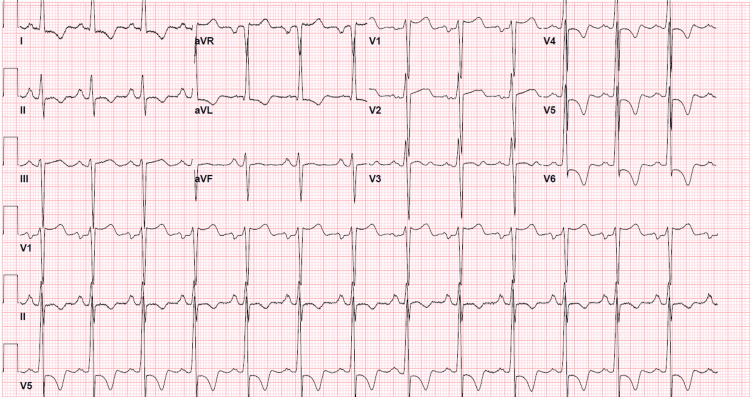
Initial EKG Initial EKG was significant for left ventricular hypertrophy with early repolarization abnormalities, persistent ST depressions with T-wave inversions in leads V4-V6, and concave ST elevations in leads aVR and V1. EKG: electrocardiogram

Initial laboratory workup focused on evaluating potential end-organ damage as well as investigating possible etiologies of secondary hypertension. Complete blood count (CBC) with differential was pan-normal. Basic metabolic panel (BMP) was remarkable for a blood urea nitrogen (BUN) of 25 mg/dL (ref: 7-20) and creatinine of 2.86 mg/dL (ref: 0.70-1.30). This led to an estimated glomerular filtration rate of 27 ml/min/1.73m^2^. High-sensitivity troponin was trended and peaked at 78 ng/L (ref: <35). Electrocardiogram (EKG) (Figure 1) revealed left ventricular hypertrophy with early repolarization abnormalities, persistent ST depressions with T-wave inversions in leads V4-V6, and concave ST elevations in leads aVR and V1.

**Figure 2:**
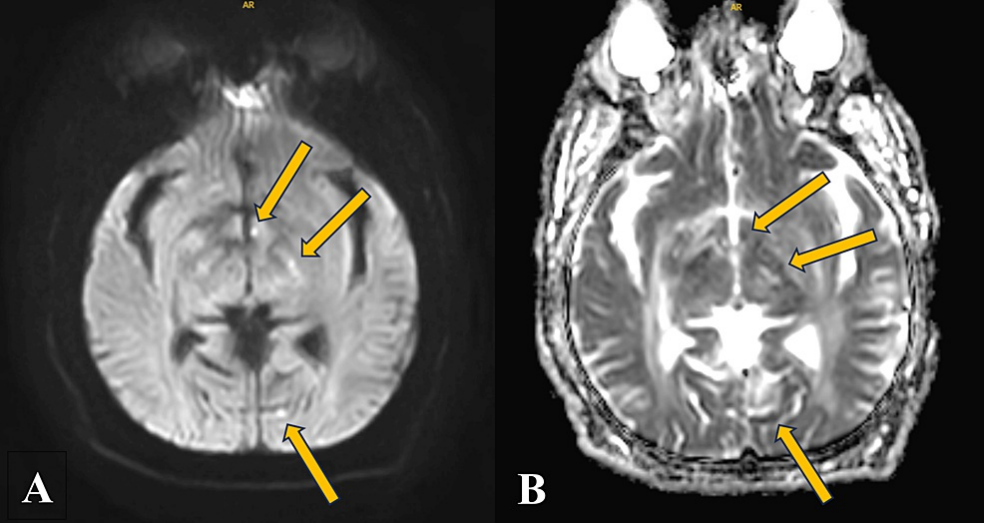
ADC MRI series ADC MRI series revealing multiple punctate hypointensities consistent with ischemic changes. ADC: apparent diffusion coefficient

**Figure 3:**
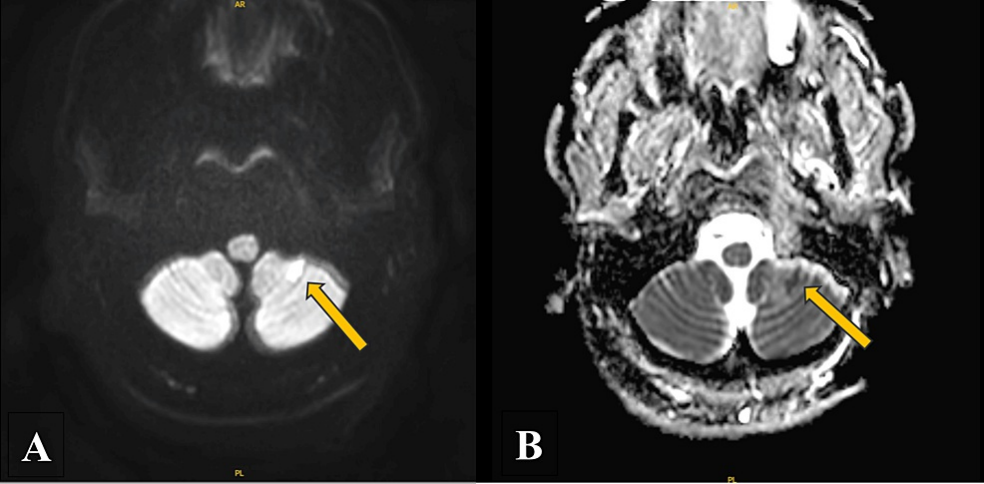
DWI MRI series DWI MRI series revealing a hypointense foci consistent with a recent small left cerebellar infarction. DWI: diffusion-weighted imaging

Given his severe hypertension with neurological complaints, a CT of the head without contrast was obtained. This revealed extensive age-indeterminate hypodensities involving the bilateral cerebral white matter as well as the bilateral deep gray nuclei. Our radiology colleagues noted that this is markedly abnormal and that further evaluation with an MRI of the brain with and without contrast was advised for further characterization. The MRI of the brain as well as MRA of the head and neck was obtained and revealed small recent infarcts observed at the level of the left cerebellum, left occipital lobe, and left hypothalamus with extensive chronic ischemic changes and no significant stenoses (Figure 2 and Figure 3).

At this point, neurology was consulted and recommended aspirin 81 mg daily and atorvastatin 40 mg daily. Additionally, they recommended a workup for his secondary hypertension and multiple infarcts which included checking lupus anticoagulant, B2GI, and anti-cardiolipin antibody. The results of these tests can be seen in Table 1.

**Table 1:** APS antibody panel Results of the APS antibody panel were unremarkable with the exception of an elevated IgA B2GI antibody level. APS: antiphospholipid syndrome; B2GI: beta-2 glycoprotein I; LA: lupus anticoagulant; aPTT: activated partial thromboplastin time

Test	Result	Reference range
Anti-cardiolipin antibody		
IgG	<9 U/mL	0-14 U/mL
IgM	<9 U/mL	0-12 U/mL
B2GI antibody		
IgG	<9 U	0-20 U
IgA	59 U	0-25 U
IgM	11 U	0-32 U
LA		
PTT-LA	50.8 sec	0-43.5 sec
dRVVT	50.9 sec	0-47.0 sec
Interpretation of LA test	No LA was detected. Mixing studies suggest the presence of an inhibitor. It should be noted that mixing studies performed on samples with minimally extended aPTT results can be equivocal.	

Assessment and differential diagnosis

Overall, this is a case of a male in his mid-40s presenting with hypertensive urgency/emergency. He was found to have markedly abnormal chronic ischemic changes on brain imaging. Laboratory testing was significant for elevated IgA B2GI. The differential is fairly broad and can be initially split into two categories: primary versus secondary hypertension. Primary hypertension is possible, although less likely to cause blood pressures as high as in this individual. Secondary hypertension includes renal artery stenosis, other kidney disease, hyperaldosteronism, (subclinical) APS, cardiac disease, and more. In this case, the APS differential diagnosis can be thought of as "subclinical," as the patient technically did not meet the diagnostic criteria for the disease, prompting the rationale behind this case report.

Treatment

Anti-hypertensives were started and slowly titrated to a target systolic blood pressure of less than 140 mmHg. This regimen included hydralazine 100 mg every 12 hours, labetalol 200 mg every 12 hours, nifedipine 120 mg daily, and spironolactone 50 mg daily. Additionally, he was started on aspirin 81 mg daily and atorvastatin 40 mg daily.

Outcome and follow-up

Although the patient had difficulty adhering to his medication regimen, his blood pressure was controlled at three-month follow-up. Additionally, subsequent head imaging revealed stable chronic ischemic changes without any significant interval changes. This likely indicates a degree of success in preventing subsequent thromboses.

## Discussion

This case report discusses a difficult-to-treat hypertensive emergency and non-specific neurologic symptoms that led to the discovery of increased IgA B2GI. Nearly 120 million adults in the United States are diagnosed with hypertension, while 805,000 people suffer from a myocardial infarction (MI) every year [3]. Nearly 800,000 people suffer from strokes every year [4]. Hypertension, MI, and strokes occur at higher rates in non-Hispanic black adults and Pacific Islander populations as compared to white adults [3-4]. Several key risk factors for MI and strokes have been well studied including hypertension, hyperlipidemia, smoking, diabetes mellitus, and obesity [4]. However, the impact of certain antibodies on these sequelae remains partially unknown. Thus, given the high incidence of hypertension, stroke, and MI, gaining a deeper understanding of this pathophysiology could positively impact preventative medicine and even outcomes.

B2GI is a unique protein that has multiple functions throughout the body, with roles involving coagulation and complement. The protein can form IgA, IgM, and IgG complexes that can be detected in patients with APS [1]. APS is a complex autoimmune condition in which patients have thrombotic and/or obstetrical events with persistently elevated antiphospholipid antibodies. Non-thrombotic complications can also occur such as livedo reticularis, heart valve thickening, valvular vegetations, and hypertension [5]. Thrombotic events include both venous and arterial thromboembolism. Obstetrical morbidity includes recurrent fetal death after 10 weeks, preeclampsia, or placental insufficiency causing intrauterine growth restriction [6]. Although there is a clear association between APS and microvascular events, a clear association between macrovascular events such as stroke and MI is not currently established. Laboratory criteria must be met for the diagnosis of APS which includes present IgG or IgM isotype of anti-cardiolipin antibodies, anti-B2GI antibodies, and lupus anticoagulant [7]. The international criteria for the classification of APS can be found in Table 2 [2]. 

**Table 2:** International criteria for the classification of APS At least one clinical criteria and one laboratory criteria must be met to make the diagnosis of APS. In the case of fetal morbidity/mortality, the fetus must be morphologically normal, and alternative anatomic, hormonal, genetic, and chromosomal causes should be ruled out. Lastly, all laboratory criteria must be present on two or more occasions at least 12 weeks apart. APS: antiphospholipid syndrome; B2GI: beta-2 glycoprotein I

Clinical criteria	
Criteria	Description
Vascular thrombosis	One or more instances of confirmed venous or arterial thrombosis
Pregnancy morbidity/mortality	One of more instances of fetal demise after the 10th week of gestation, OR
	One or more instances of birth prior to the 34th week of gestation due to eclampsia/preeclampsia or placental insufficiency, OR
	Three or more unexplained consecutive spontaneous abortions prior to the 10th week of gestation
Laboratory criteria	
Lupus anticoagulant	-
Anti-cardiolipin antibody	IgG and/or IgM isotypes
B2GI antibody	IgG and/or IgM isotypes

Testing should generally be limited to patients under 50 years old who present with a(n) unprovoked thromboembolism(s) or unusual site thrombosis [7]. Unusual sites can include the portal vein, mesenteric veins, and most arterial sites. Women with recurrent second- and third-trimester pregnancy loss and thromboembolisms also warrant a workup for APS [7]. Laboratory testing should be delayed until after the resolution of the acute event and must be persistently positive 12 weeks apart to meet the diagnostic criteria [7]. 

The treatment of APS utilizes risk stratification and is aimed at limiting thromboembolic events. Risk stratification is based on age, positive antibodies, concurring risk factors for thrombosis, and other concurrent autoimmune diseases [8]. Primary/secondary thrombotic prevention is dependent on the annual risk of a thrombotic event. Daily low-dose aspirin has minimal evidence of efficacy in the treatment of APS but is often used in patients with concurrent cardiovascular disease [8]. With insufficient evidence of the efficacy of aspirin in primary prevention, more research is needed to determine optimal prevention strategies. Secondary venous and arterial thrombosis is often initially treated with unfractionated or low molecular weight heparin and transitioned to long-term warfarin treatment [8]. Data on direct thrombin inhibitor use is limited. Direct oral anticoagulants (DOACs; factor Xa inhibitors) such as apixaban and rivaroxaban have shown worse outcomes compared to warfarin as seen in the ASTRO-APS study [1,9]. Statins should also be simultaneously initiated to reduce endothelial cell activation [9].

B2GI is a complement control protein within circulation and contains a target of autoantibodies in APS [1]. In vitro studies have shown that B2GI has both pro- and anticoagulant factors [1]. In vivo, B2GI inhibits activated protein C, a protein that inhibits tissue factor pathway [1]. The binding of B2GI on cell surfaces upregulates E-selectin and tissue factor, both being prothrombotic molecules [8]. Testing for B2GI antibodies has classically been directed at IgM or IgG isotypes. However, many newer assays now include the IgA isotype.

Although the IgA isotype is tested for, it is currently not included in the laboratory diagnostic criteria of APS but may still hold clinical significance as seen in this patient. A newer notion of seronegative APS can be seen in patients who present with clinical manifestations of APS but do not meet laboratory criteria for APS diagnosis [10]. Pierangeli et al. have shown that patients with elevated IgA B2GI antibodies have the potential to develop APS and are associated with obstetric complications and thrombosis [1,10-11]. Thus, there is a growing urge to include IgA positivity in the criteria for APS diagnosis [12]. Future studies should consider further investigations into the effects of IgA B2GI and its qualification in the criteria for the diagnosis of APS.

## Conclusions

APS is a nuanced disease that lies at the crossroads of rheumatology and benign hematology. If left undiagnosed or untreated, its implications can be catastrophic, causing venous and arterial thromboses as well as pregnancy complications. The pathophysiology of APS is detailed and remains partially unknown. Although anti-B2GI plays a role in APS, the diagnostic criteria does not include the IgA isotype, which is often tested along with the other APS antibodies. Thus, the IgA isotype may play a role in thrombosis and APS-like symptoms while remaining overlooked in diagnostic workups. Future research in this area should be aimed at quantifying the impact of the IgA isotype of anti-B2GI versus its other isotypes and even those of anti-cardiolipin antibodies.
